# Associations between dietary carotenoid intakes and the risk of depressive symptoms

**DOI:** 10.29219/fnr.v64.3920

**Published:** 2020-12-28

**Authors:** Honghan Ge, Tingting Yang, Jing Sun, Dongfeng Zhang

**Affiliations:** 1 Department of Epidemiology and Health Statistics, Qingdao University Medical College, Qingdao, China; 2 Department of Hospital-Acquired Infection Control, The Affiliated Hospital of Qingdao University, Qingdao, China

**Keywords:** depressive symptoms, dietary carotenoid intakes, restricted cubic spline models, dose–response relationship, NHANES

## Abstract

**Background:**

Dietary factors play an important role in the development of depressive symptoms. Carotenoids have effective antioxidant and anti-inflammatory effects, but few studies have explored the associations between dietary carotenoid intake and depressive symptoms.

**Objective:**

To evaluate the association between dietary carotenoid intake and the risk of depressive symptoms in adults from the United States.

**Design:**

This cross-sectional study included adult participants from the National Health and Nutrition Examination Survey 2009–2016. Depressive symptoms were assessed using the Patients’ Health Questionnaire-9. Intake of carotenoids was obtained through two 24-h dietary recall interviews. We applied logistic regression models and restricted cubic spline models to evaluate the associations of dietary alpha-carotene, beta-carotene, beta-cryptoxanthin, lycopene, lutein with zeaxanthin, and total carotenoid intake with the risk of depressive symptoms.

**Results:**

Overall, a total of 17,401 adults aged 18–80 years were included in this study. After adjustment for potential confounders, the odds ratios (95% confidence intervals) of depressive symptoms in the highest versus lowest quartiles were 0.71 (0.56–0.92) for alpha-carotene, 0.59 (0.47–0.75) for beta-carotene, 0.71 (0.55–0.92) for beta-cryptoxanthin, 0.66 (0.49–0.89) for lycopene, 0.50 (0.39–0.64) for lutein with zeaxanthin, and 0.59 (0.45–0.78) for total carotenoid intake. U-shaped dose–response relationships were found between both beta-carotene and lutein with zeaxanthin intake and the risk of depressive symptoms.

**Conclusion:**

Results suggest that alpha-carotene, beta-carotene, beta-cryptoxanthin, lycopene, lutein with zeaxanthin, and total carotenoid intake may be inversely associated with the risk of depressive symptoms in the U.S. adults.

## Popular scientific summary

- Higher intakes of carotenoid were protective against depressive symptoms.
- This study evaluated the associations between dietary intakes of five kinds of carotenoids and total carotenoid with depressive symptoms.
- Our findings may be helpful to develop dietary guidelines for preventing depressive symptoms.

According to the World Health Organization, more than 300 million people experience depression globally (1). In the United States, the prevalence of depression was 5.9% in 2015 (2), and the financial burden of depression in the United States was $188 billion in 2012 (3). By 2030, depression is calculated to be the second leading cause of disability-adjusted life years worldwide and the first leading cause in high-income countries (4). Although there have been effective treatments for depression, the rates of treatments are low (1). Therefore, it is important to explore the risk factors and preventive measures for depression.

The pathogenesis of depression remains unclear. The etiology of depression is related to genetic and social environmental factors (5–8). In recent years, several dietary factors, such as fish (9), coffee (10), fruit and vegetable (11), dietary fiber (12), magnesium (13), zinc, iron, copper, and selenium intakes, have been found to be associated with the risk of depression (14). These dietary factors play a protective role in the development of depression through their anti-inflammatory or antioxidant properties. Additionally, carotenoids are also known to have effective antioxidant and anti-inflammatory effects (15, 16).

Previous studies have found that carotenoids could reduce the risk of many diseases, such as cancer, coronary vascular disease, ocular disease, hypertension, and diabetes (17–21). Some epidemiologic studies have also reported an association between carotenoid intake and the risk of depression (22–26). A cross-sectional study found that higher intakes of alpha-carotene and beta-carotene were inversely associated with the risk of depressive symptoms among midlife women in the United States (22). A case–control study also suggested an inverse association between beta-carotene intake and the risk of depression in Korean students (24). Another cross-sectional study among American elderly showed that the risk of depression was significantly associated with dietary cryptoxanthin intake, but not with four other carotenoids and total carotenoid intakes (25). However, these studies were conducted only for specific populations and the results were not consistent. Therefore, using data from the National Health and Nutrition Examination Survey (NHANES) 2009–2016, we evaluated the associations between dietary carotenoid intakes and the risk of depressive symptoms in the U.S. adults in this study.

## Materials and methods

### Study population

**Fig. 1:**
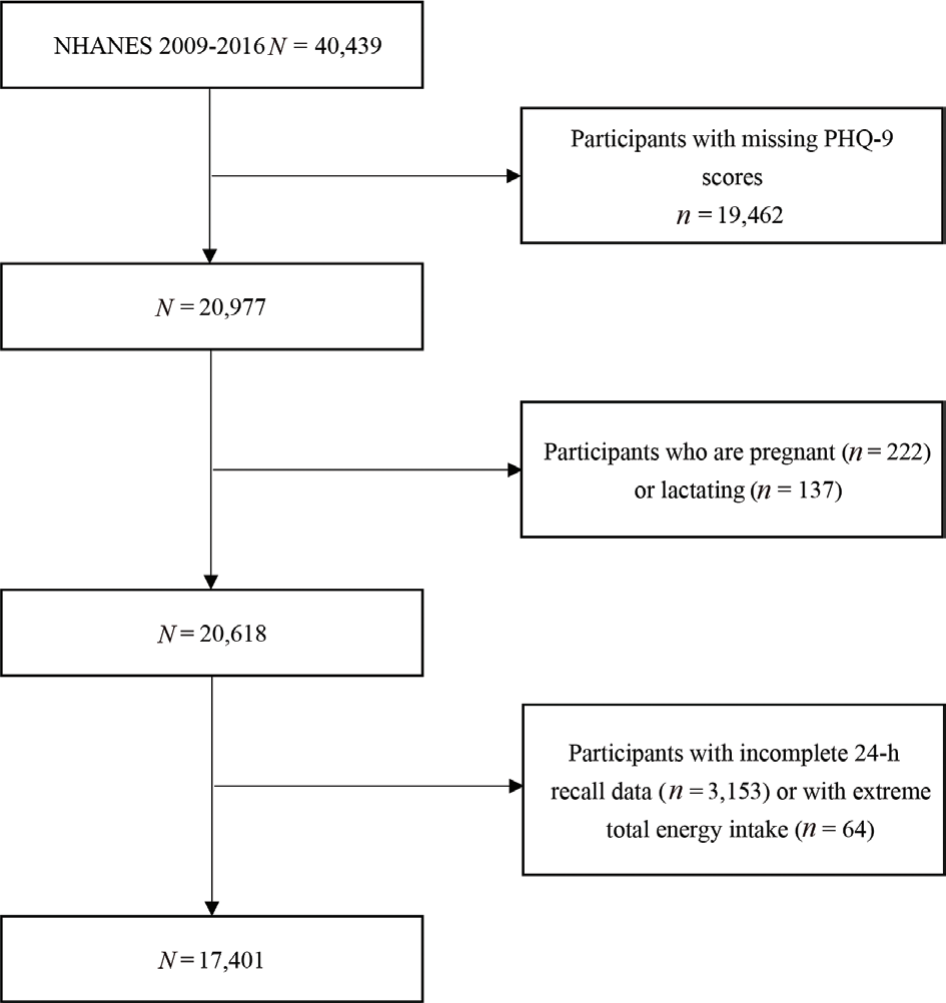
Flow chart of the screening process for the selection of eligible participants.

The NHANES is aimed at assessing the health and nutritional status of adults and children (aged 0–80 years) in the United States (27). In this study, we used data from NHANES 2009–2010, 2011–2012, 2013–2014, and 2015–2016. Of the four cycles, there were 40,439 participants, where only 20,977 of them completed the depression questionnaire. Furthermore, we excluded those who were pregnant (*n* = 222) and lactating (*n* = 137). In addition, 3,153 participants were also excluded because their 24-h dietary recall data were not complete (those who did not finish the two 24-h dietary recall interviews). Moreover, individuals were omitted whose total energy intake was <500 or >5,000 kcal/day for female, and <500 or >8,000 kcal/day for male (*n* = 64). Finally, a total of 17,401 participants (8,555 men and 8,847 women) were included in this study (Fig. 1).

### Assessment of depressive symptoms

Depressive symptoms were assessed using a nine-item depressive symptoms screening instrument, Patient’s Health Questionnaire-9 (PHQ-9). Each item scored from 0 to 3, and the total score ranged from 0 to 27. Participants were considered to have depressive symptoms if their PHQ-9 scores were ≥10 (28).

### Dietary intake assessment

Dietary carotenoid intakes were obtained through two 24-h dietary recall interviews. The first dietary recall interview was collected at the Mobile Examination Center (MEC), and the second interview was conducted by telephone 3–10 days later. The 24-h dietary recall interview has been proven to be an effective method for assessing the intake of nutrients and energy (29, 30). The food composition database used in NHANES was the United States Department of Agriculture (USDA) Dietary Sources of Nutrients database (31). In our study, the intake of alpha-carotene, beta-carotene, beta-cryptoxanthin, lycopene, and lutein with zeaxanthin was measured. As such, we defined the sum of the aforementioned five carotenoids as the total carotenoid. Dietary carotenoid intakes were defined as the average dietary intake data of the two 24-h dietary recall interviews.

### Covariates

The following covariates were included in this study: age (18–39, 40–59, and 60–80 years), gender (male and female), ethnicity (Mexican American, other Hispanic, non-Hispanic white, non-Hispanic black, and other ethnicity; ethnicity was included in the model as a dummy variable, with Mexican American as the reference group), educational level (below high school, high school, and above high school), body mass index (BMI) (<25, 25 to <30, and ≥30 kg/m**^2^**), annual family income (under $20,000 and $20,000 and over), work physical activity and recreational physical activity, hypertension (yes/no), diabetes (yes/no), smoking status (smoking at least 100 cigarettes in life or not), drinking status (had at least 12 alcohol drinks a year or not), and energy intake. Total energy intake was obtained from 24-h dietary recall interviews (13).

### Statistical analysis

Characteristics were described as mean ± standard deviation for normal variables, median (interquartile range) for non-normal variables, and number (percentage) for categorical variables. The Kolmogorov–Smirnov normality test was used to verify the normality of carotenoid and energy intakes. Student’s *t*-tests would be applied when normality assumptions were satisfied; otherwise, the equivalent non-parametric test would be used. The Chi-square test was used to examine the difference in the percentage of categorical variables between participants with or without depressive symptoms.

Carotenoid intakes were categorized by quartiles (quartile 1: <25th; quartile 2: 25 to <50th; quartile 3: 50 to <75th; quartile 4: ≥75th). Depressive symptoms were analyzed as a binary variable. Subjects with a PHQ-9 score ≥10 were defined as the case group, whereas those with a PHQ-9 score less than 10 were defined as the control group. The odds ratios (ORs) (95% confidence intervals [CIs]) were estimated by conducting binary logistic regression analyses to test the associations between quartiles of carotenoid and the presence of depressive symptoms, with quartile 1 as the reference category. Model 1 was adjusted for age and gender. Model 2 was adjusted for age, gender, ethnicity, educational level, BMI, annual family income, work physical activity, recreational physical activity, hypertension, diabetes, smoking status, drinking status, and total energy intake. Considering that those with higher body weight may have more nutrients intake, we adjusted dietary carotenoid intake by weight to conduct a sensitivity analysis. In addition, stratified analyses were performed based on gender (male or female), age (18–39, 40–59, and 60 years old and over), and ethnicity (Mexican American, other Hispanic, non-Hispanic white, non-Hispanic black, and other ethnicity). We also assessed the dose–response relationship using restricted cubic spline with three knots at the 5th, 50th, and 95th. The adjusted covariates in the restricted cubic spline were the same as the covariates adjusted in Model 2 of the logistic regression. We tested the value of the second zero spline coefficient to calculate the non-linear *P*-value. A two-sided *P*-value <0.05 was considered statistically significant. A new 8-year dietary weight was created by taking one-fourth of the 2-year weight following the NHANES guidelines (32). All analyses were conducted using STATA 15.0 (Stata Corp., College Station, TX, USA) and SPSS 18.0 (SPSS Inc., Chicago, IL, USA).

## Results

Table 1 presents the characteristics of the selected population based on depressive symptoms status. As such, the prevalence of depressive symptoms was 8.88% for all participants. The prevalence of depressive symptoms was higher in females, smokers, and those with hypertension and diabetes. Compared with participants without depressive symptoms, those with depressive symptoms tended to be younger and obese. In addition, individuals with depressive symptoms were more likely to have lower educational level, family income, work activity, and recreational activity. Alpha-carotene, beta-carotene, beta-cryptoxanthin, lycopene, lutein with zeaxanthin, total carotenoid, and energy intake in participants with depressive symptoms were significantly lower than those without depressive symptoms.

**Table 1:** Characteristics of participants by depressive symptoms, National Health and Nutrition Examination Survey 2009–2016 (*N* = 17,401)

	Without depressive symptoms	With depressive symptoms	*P*
Number of subjects (%)^a^	15,856 (91.12)	1,545 (8.88)	
Age (year) (%)^a^			<0.01
18–39	5,640 (35.57)	495 (32.04)	
40–59	5,019 (31.65)	606 (39.22)	
60–80*	5,197 (32.78)	444 (28.74)	
Gender (%)^a^			<0.01
Male	8,002 (50.47)	553 (35.79)	
Female	7,854 (49.53)	992 (64.21)	
Ethnicity (%)^a^			<0.01
Mexican American	2,316 (14.61)	232 (15.02)	
Other Hispanic	1,571 (9.91)	214 (13.85)	
Non-Hispanic white	6,781 (42.77)	648 (41.94)	
Non-Hispanic black	3,380 (21.32)	333 (21.55)	
Other ethnicity	1,808 (11.40)	118 (7.64)	
Educational level (%)^a^			<0.01
<High school	3,363 (21.24)	526 (34.07)	
High school	3,662 (23.12)	370 (23.96)	
>High school	8,811 (55.64)	648 (41.97)	
Body mass index (BMI) (%)^a^			<0.01
<25 kg/m^2^	4,656 (29.63)	373 (24.48)	
25 to <30 kg/m^2^	5,201 (33.10)	367 (24.08)	
≥30 kg/m^2^	5,856 (37.27)	784 (51.44)	
Family income (%)^a^			<0.01
Under $20,000	3,431 (22.59)	656 (44.35)	
$20,000 and over	11,758 (77.41)	823 (55.65)	
Work activity (%)^a^			0.025
Vigorous	2,980 (18.80)	294 (19.05)	
Moderate	3,411 (21.52)	287 (18.60)	
Other	9,459 (59.68)	962 (62.35)	
Recreational activity (%)^a^			<0.01
Vigorous	3,850 (24.29)	178 (11.52)	
Moderate	4,349 (27.44)	323 (20.91)	
Other	7,653 (48.27)	1,044 (67.57)	
Hypertension (%)^a^	5,519 (38.84)	731 (37.38)	<0.01
Diabetes (%)^a^	1,911 (12.06)	316 (20.51)	<0.01
Smoke at least 100 cigarettes in life (%)^a^	6,467 (41.98)	891 (58.73)	<0.01
Had at least 12 alcohol drinks a year (%)^a^	11,178 (71.53)	1,116 (73.13)	0.096
Total energy (kcal/day)^b^	1921.50 (983)	1807.50 (1,018)	<0.01
Alpha-carotene (mcg/day)^b^	87.50 (444)	48.5 (269)	<0.01
Beta-carotene (mcg/day)^b^	1112.50 (2323.50)	669 (1530.50)	<0.01
Beta-cryptoxanthin (mcg/day)^b^	43 (87)	30 (68.50)	<0.01
Lycopene (mcg/day)^b^	2,520 (5694.25)	2116.50 (4827.75)	<0.01
Lutein with zeaxanthin (mcg/day)^b^	843 (1,154)	625 (772.25)	<0.01
Total carotenoid (mcg/day)^b^	6543.50 (9235.25)	4,865 (7948.75)	<0.01

Data are number of subjects (percentage) or medians (interquartile ranges).

a Chi-square test is used to compare the percentage between participants with and without depressive symptoms.

b Mann–Whitney U test is used to compare the mean values between participants with and without depressive symptoms.

* Individuals aged 80 and over are topcoded at 80 years of age.

Table 2 presents results of the logistic regression analyses. The crude ORs with 95% CI of depressive symptoms in the highest versus lowest quartiles were 0.53 (0.43–0.65) for alpha-carotene, 0.41 (0.34–0.50) for beta-carotene, 0.54 (0.43–0.69) for beta-cryptoxanthin, 0.57 (0.44–0.74) for lycopene, 0.33 (0.27–0.42) for lutein with zeaxanthin, and 0.41 (0.32–0.52) for total carotenoid. After adjustment for age and gender in Model 1, the results remained statistically significant. In Model 2, the multivariate-adjusted ORs with 95% CI of depressive symptoms were 0.71 (0.56–0.92) for alpha-carotene, 0.59 (0.47–0.75) for beta-carotene, 0.71 (0.55–0.92) for beta-cryptoxanthin, 0.66 (0.49–0.89) for lycopene, 0.50 (0.39–0.64) for lutein with zeaxanthin, and 0.59 (0.45–0.78) for total carotenoid. Similar results were found in the sensitivity analysis, in which dietary carotenoid intake was adjusted by weight (Supplementary Table S1).

**Table 2:** Weighted odds ratios (95% confidence intervals) of depressive symptoms across quartiles of carotenoid (mcg) intake, National Health and Nutrition Examination Survey 2009–2016 (*N* = 17,401)

	Case/participants	Crude^a^	Model 1^a^	Model 2^a^
Alpha-carotene (mcg/day)				
<24	537/4,381	1.00 (ref.)	1.00 (ref.)	1.00 (ref.)
24 to <82.5	376/4,328	0.63 (0.52–0.76)**	0.64 (0.53–0.77)**	0.77 (0.63–0.95)*
82.5 to <451.5	332/4,346	0.60 (0.48–0.74)**	0.59 (0.47–0.74)**	0.71 (0.55–0.92)*
≥451.5	300/4,347	0.53 (0.43–0.65)**	0.53 (0.42–0.66)**	0.71 (0.56–0.92)*
Beta-carotene (mcg/day)				
<427.38	579/4,355	1.00 (ref.)	1.00 (ref.)	1.00 (ref.)
427.38 to <1060.5	390/4,350	0.60 (0.49–0.73)**	0.61 (0.50–0.76)**	0.65 (0.51–0.83)**
1060.5 to <2693.88	305/4,347	0.45 (0.36–0.56)**	0.45 (0.35–0.56)**	0.54 (0.42–0.70)**
≥2693.88	271/4,350	0.41 (0.34–0.50)**	0.41 (0.33–0.50)**	0.59 (0.47–0.75)**
Beta-cryptoxanthin (mcg/day)				
<14.5	484/4,462	1.00 (ref.)	1.00 (ref.)	1.00 (ref.)
14.5 to <41.5	417/4,256	0.91 (0.72–1.15)	0.91 (0.72–1.16)	1.09 (0.84–1.41)
41.5 to <99.5	354/4,348	0.67 (0.54–0.83)**	0.67 (0.54–0.84)**	0.77 (0.59–0.99)*
≥99.5	290/4,336	0.54 (0.43–0.69)**	0.56 (0.44–0.71)**	0.71 (0.55–0.92)*
Lycopene (mcg/day)				
<745.5	457/4,352	1.00 (ref.)	1.00 (ref.)	1.00 (ref.)
745.5 to <2,484	383/4,350	0.83 (0.68–1.00)	0.82 (0.67–1.00)	0.87 (0.70–1.08)
2,484 to <6364.5	388/4,350	0.92 (0.72–1.19)	0.92 (0.71–1.19)	1.04 (0.77–1.39)
≥6364.5	317/4,350	0.57 (0.44–0.74)**	0.61 (0.47–0.80)**	0.66 (0.49–0.89)*
Lutein with zeaxanthin (mcg/day)				
<449	555/4,353	1.00 (ref.)	1.00 (ref.)	1.00 (ref.)
449 to <820.25	420/4,348	0.72 (0.59–0.89)**	0.75 (0.60–0.92)**	0.89 (0.70–1.14)
820.25 to <1,571	329/4,352	0.50 (0.39–0.64)**	0.53 (0.41–0.67)**	0.63 (0.49–0.81)**
≥1,571	241/4,349	0.33 (0.27–0.42)**	0.33 (0.27–0.42)**	0.50 (0.39–0.64)**
Total carotenoid (mcg/day)				
<3,088	548/4,351	1.00 (ref.)	1.00 (ref.)	1.00 (ref.)
3,088 to <6,380	383/4,351	0.69 (0.55–0.86)**	0.71 (0.57–0.89)**	0.78 (0.62–0.99)*
6,380 to <12,179	335/4,350	0.58 (0.46–0.72)**	0.58 (0.47–0.73)**	0.71 (0.56–0.89)**
≥12,179	279/4,350	0.41 (0.32–0.52)**	0.43 (0.34–0.55)**	0.59 (0.45–0.78)**

a Calculated using binary logistic regression. Model 1 adjusted for age and gender. Model 2 adjusted for age and gender, ethnicity, educational level, BMI, annual family income, work activity, recreational activity, hypertension, diabetes, smoking status, drinking status, and total energy intake.

* *P* < 0.05;

** *P* < 0.01.

Table 3 shows the associations between total dietary carotenoid intake and the risk of depressive symptoms in stratified analyses by age and gender. In stratified analyses by age, the inverse association between total carotenoid intake and the risk of depressive symptoms was significant in the third versus the first quartile analysis in all three models among participants aged 18–39 years. For the highest quartile, the inverse association was significant in the unadjusted model and Model 1. For participants aged 40–59 years, all levels of total carotenoid intakes were associated with a decreased risk of depressive symptoms in the unadjusted model and Model 1. In Model 2, the association was significant only in the highest quartile of total dietary carotenoid intake. For participants aged 60 years and older, the inverse associations with depressive symptoms were significant in the highest quartile of dietary total carotenoid intake in three models. The ORs (95% CIs) of depressive symptoms in the highest quartile of total carotenoid intake in Model 2 were 0.65 (0.44–0.97) and 0.34 (0.22–0.54) for participants aged 40–59 years and 60 years and older, respectively. In stratified analyses by gender, the inverse association between the highest quartile of total carotenoid and the risk of depressive symptoms was significant in all three models. For the third quartile of total carotenoid intake, the association was significant in unadjusted model and Model 1. In females, all levels of dietary total carotenoid intakes were associated with a decreased risk of depressive symptoms in the three models. The ORs (95% CIs) of depressive symptoms in the highest quartile of total carotenoid intake in Model 2 were 0.56 (0.37–0.84) and 0.62 (0.44–0.88) for males and females, respectively.

**Table 3:** Weighted odds ratios (95% confidence intervals) of depressive symptoms across quartiles of dietary total carotenoid intake, stratified by age and gender, National Health and Nutrition Examination Survey 2009–2016 (*N* = 17,401)

Total carotenoid	Crude^a^	Model 1^a^	Model 2^a^
18–39 years old			
<3,088	1.00 (ref.)	1.00 (ref.)	1.00 (ref.)
3,088 to <6,380	0.74 (0.50–1.10)	0.78 (0.53–1.15)	0.74 (0.49–1.13)
6,380 to <12,179	0.53 (0.38–0.73)**	0.54 (0.39–0.76)**	0.58 (0.41–0.82)**
≥12,179	0.51 (0.34–0.78)**	0.55 (0.36–0.84)**	0.67 (0.41–1.04)
40–59 years old			
<3,088	1.00 (ref.)	1.00 (ref.)	1.00 (ref.)
3,088 to <6,380	0.62 (0.47–0.83)**	0.65 (0.49–0.87)**	0.80 (0.56–1.14)
6,380 to <12,179	0.61 (0.45–0.83)**	0.62 (0.46–0.85)**	0.87 (0.62–1.23)
≥12,179	0.39 (0.29–0.40)**	0.42 (0.31–0.58)**	0.65 (0.44–0.97)*
≥60 years old			
<3,088	1.00 (ref.)	1.00 (ref.)	1.00 (ref.)
3,088 to <6,380	0.71 (0.46–1.12)	0.71 (0.46–1.11)	0.72 (0.43–1.22)
6,380 to <12,179	0.56 (0.30–1.02)	0.57 (0.31–1.05)	0.59 (0.32–1.09)
≥12,179	0.27 (0.18–0.41)**	0.28 (0.18–0.43)**	0.34 (0.22–0.54)**
Male			
<3,088	1.00 (ref.)	1.00 (ref.)	1.00 (ref.)
3,088 to <6,380	0.86 (0.59–1.25)	0.85 (0.58–1.24)	0.99 (0.66–1.47)
6,380 to <12,179	0.59 (0.38–0.90)*	0.58 (0.38–0.89)*	0.72 (0.45–1.17)
≥12,179	0.41 (0.28–0.60)**	0.41 (0.28–0.59)**	0.56 (0.37–0.84)*
Female			
<3,088	1.00 (ref.)	1.00 (ref.)	1.00 (ref.)
3,088 to <6,380	0.63 (0.48–0.82)**	0.63 (0.48–0.82)**	0.67 (0.51–0.87)**
6,380 to <12,179	0.60 (0.46–0.78)**	0.59 (0.45–0.88)**	0.70 (0.51–0.95)*
≥12,179	0.46 (0.34–0.61)**	0.46 (0.34–0.62)**	0.62 (0.44–0.88)**

a Calculated using binary logistic regression. Model 1 adjusted for age and gender. Model 2 adjusted for age and gender, ethnicity, educational level, BMI, annual family income, work activity, recreational activity, hypertension, diabetes, smoking status, drinking status, and total energy intake.

* *P* < 0.05;

** *P* < 0.01.

The associations between total dietary carotenoid intake and the risk of depressive symptoms in stratified analyses by ethnicity are presented in Supplementary Table S2. For Mexican American, other Hispanic, and non-Hispanic black, the associations between dietary total carotenoids and depressive symptoms were no longer statistically significant, although the ORs were below 1. For non-Hispanic white and other ethnicity, total dietary carotenoid intake was inversely associated with depressive symptoms. The ORs (95% CIs) of depressive symptoms in the highest quartile of total carotenoid intake in Model 2 were 0.57 (0.39–0.85) and 0.42 (0.20–0.89) for non-Hispanic white and other ethnicity, respectively.

**Fig. 2:**
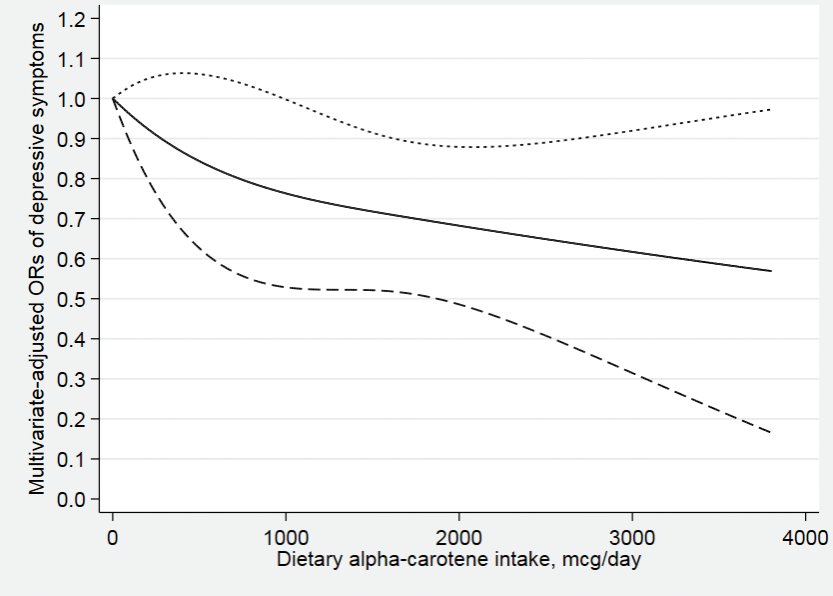
Dose–response relationship between dietary alpha-carotene intake and depressive symptoms. The association was adjusted for age, gender, ethnicity, educational level, body mass index, annual family income, work physical activity, recreational physical activity, hypertension, diabetes, smoking status, drinking status, and total energy intake. The solid line and dash line represent the estimated ORs and its 95% confidence intervals, respectively. OR, odds ratio.

**Fig. 3:**
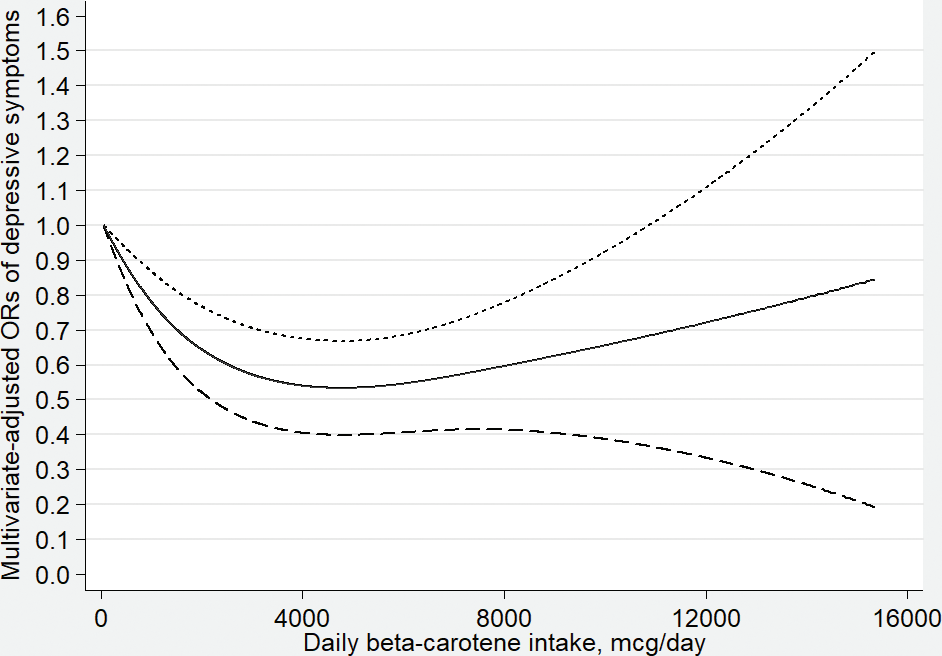
Dose–response relationship between dietary beta-carotene intake and depressive symptoms. The association was adjusted for age, gender, ethnicity, educational level, body mass index, annual family income, work physical activity, recreational physical activity, hypertension, diabetes, smoking status, drinking status, and total energy intake. The solid line and dash line represent the estimated ORs and its 95% confidence intervals, respectively. OR, odds ratio.

**Fig. 4:**
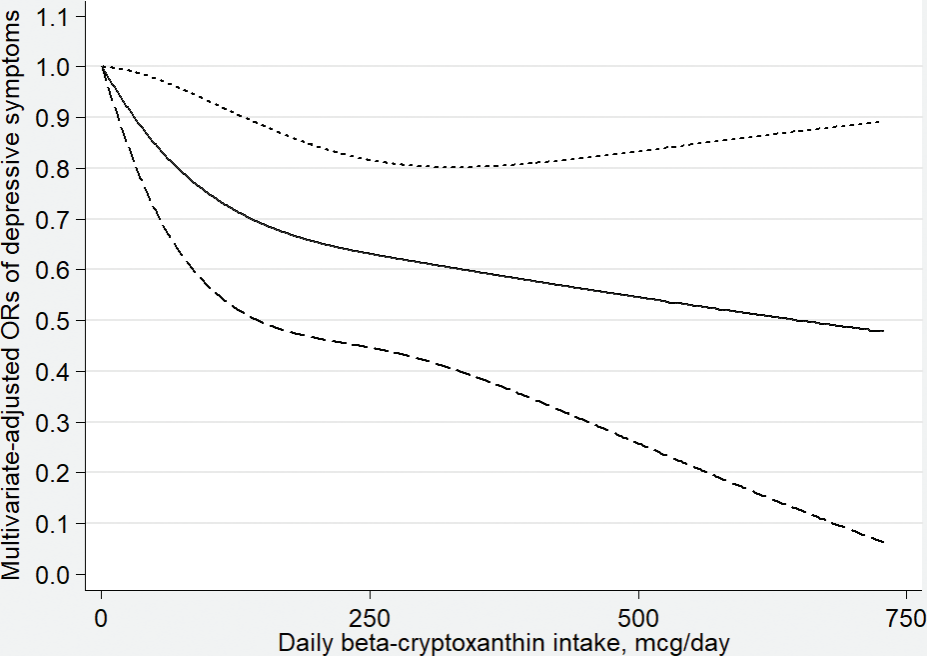
Dose–response relationship between dietary beta-cryptoxanthin intake and depressive symptoms. The association was adjusted for age, gender, ethnicity, educational level, body mass index annual family income, work physical activity, recreational physical activity, hypertension, diabetes, smoking status, drinking status, and total energy intake. The solid line and dash line represent the estimated ORs and its 95% confidence intervals, respectively. OR, odds ratio.

**Fig. 5:**
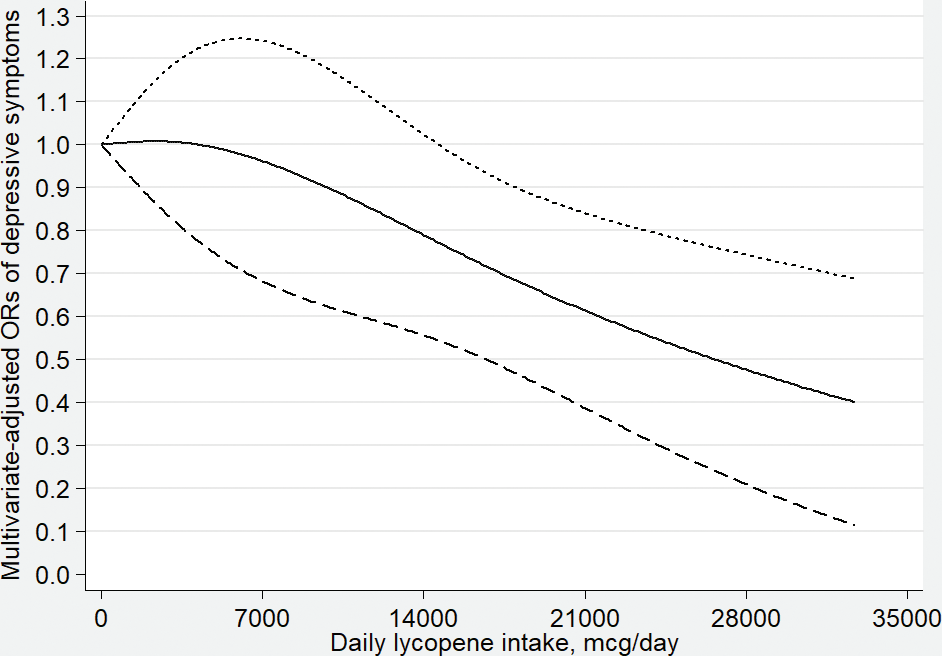
Dose–response relationship between dietary lycopene intake and depressive symptoms. The association was adjusted for age, gender, ethnicity, educational level, body mass index, annual family income, work physical activity, recreational physical activity, hypertension, diabetes, smoking status, drinking status, and total energy intake. The solid line and dash line represent the estimated ORs and its 95% confidence intervals, respectively. OR, odds ratio.

**Fig. 6:**
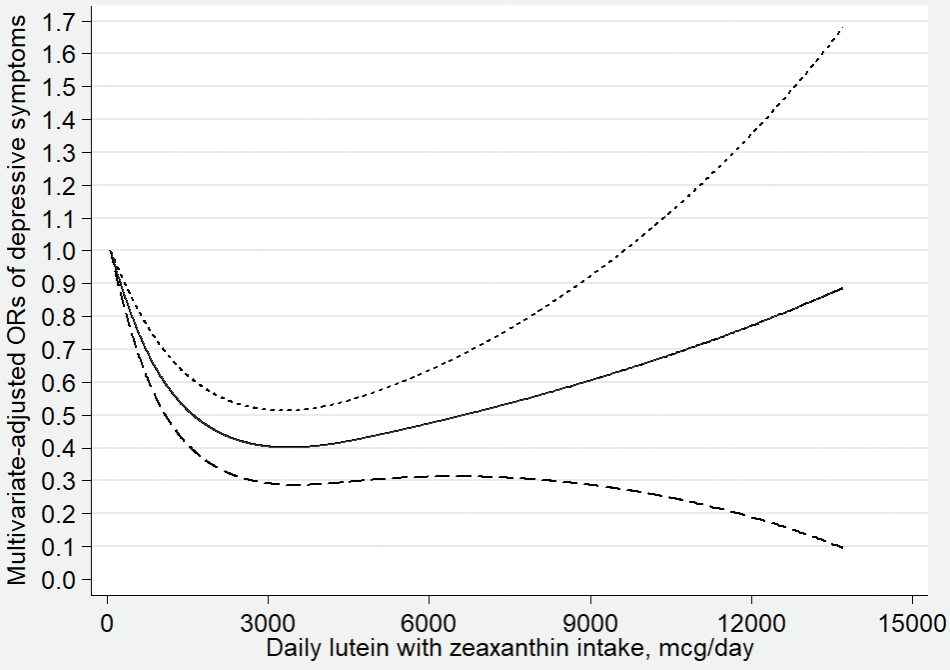
Dose–response relationship between dietary lutein with zeaxanthin intake and depressive symptoms. The association was adjusted for age, gender, ethnicity, educational level, body mass index, annual family income, work physical activity, recreational physical activity, hypertension, diabetes, smoking status, drinking status, and total energy intake. The solid line and dash line represent the estimated ORs and its 95% confidence intervals, respectively. OR, odds ratio.

**Fig. 7:**
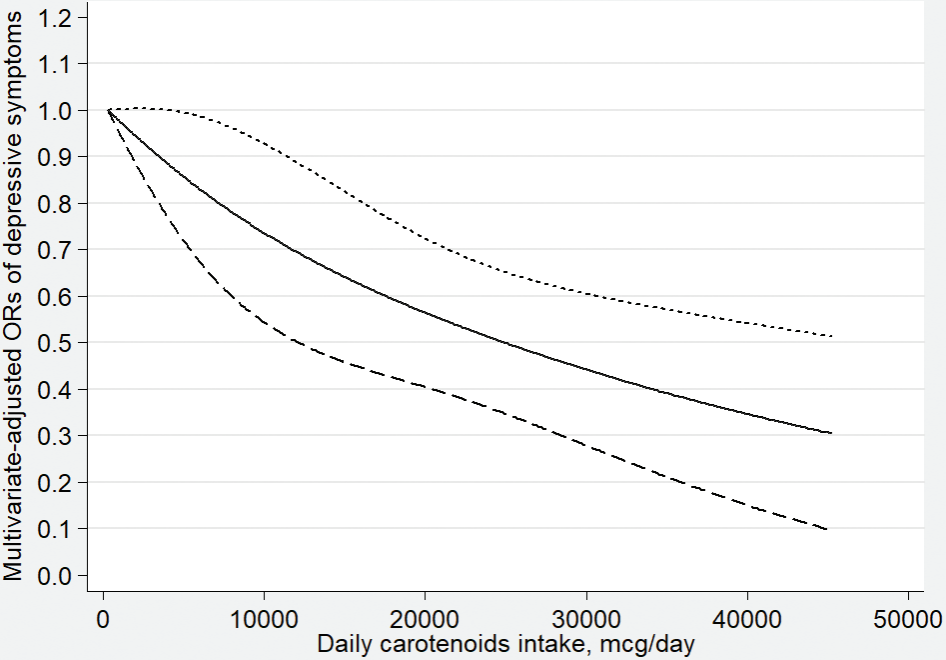
Dose–response relationship between dietary total carotenoid intake and depressive symptoms. The association was adjusted for age, gender, ethnicity, educational level, body mass index, annual family income, work physical activity, recreational physical activity, hypertension, diabetes, smoking status, drinking status, and total energy intake. The solid line and dash line represent the estimated ORs and its 95% confidence intervals, respectively. OR, odds ratio.

Figures 2–7 show the results of the dose–response relationship between depressive symptoms and dietary intakes of carotenoid. These associations were non-linear (*P*_for nonlinearity_ < 0.05). A U-shaped association was found between dietary beta-carotene intake and depressive symptoms. Similarly, the association between dietary lutein with zeaxanthin and depressive symptoms was also U shaped. There were no significant associations when alpha-carotene and lycopene intakes were lower than 960 mcg/day (OR: 0.77; 95% CI: 0.53–1.00) and 14,600 mcg/day (OR: 0.77; 95% CI: 0.54–1.00), respectively. Moreover, the associations were no longer significant when beta-carotene and lutein with zeaxanthin intakes were more than 10,810 mcg/day (OR: 0.68; 95% CI: 0.37–1.00) and 9,580 mcg/day (OR: 0.63; 95% CI: 0.27–1.00), respectively.

## Discussion

This study examined the relationship between dietary intakes of carotenoids and the risk of depressive symptoms using data from NHANES during 2009–2016. In this cross-sectional study, we found that all carotenoid intakes were inversely associated with the risk of depressive symptoms in the U.S. adults. These associations remained significant after adjustment for age, gender, and other potential confounding factors. The results of the dose-relationship analyses showed that these associations were non-linear. In stratified analyses by gender and age, the inverse association between total carotenoid intake and depressive symptoms was statistically significant among both male and female, and in the age group of 40–59 years and older than 60 years.

Similarly, a cross-sectional study indicated that higher intakes of alpha-carotene and beta-carotene were associated with a decreased risk of depressive symptoms among the U.S. midlife female (22). Park et al. (24) conducted a cross-sectional study that reported that dietary beta-carotene intake was negatively associated with depressive symptoms in Korean students. A cross-sectional study by Niu et al. (23) demonstrated that a tomato-rich diet is related to a lower prevalence of depressive symptoms among the elderly Japanese population aged ≥70 years, suggesting that dietary lycopene intake may have a beneficial effect on preventing depressive symptoms. However, Payne et al. (25) found that dietary beta-cryptoxanthin was inversely associated with depressive symptoms in older U.S. adults, while no association was found between alpha-carotene, beta-carotene, lycopene, and lutein with zeaxanthin. Meanwhile, a case–control study by Prohan et al. (26) indicated that depressive symptoms were significantly associated with dietary beta-carotene, lutein with zeaxanthin intakes, whereas associations between dietary intakes of alpha-carotene, beta-cryptoxanthin, and lycopene were not statistically significant.

To our knowledge, this is the first study to comprehensively examine the associations between dietary carotenoid intakes and depressive symptoms. There are several possible mechanisms by which carotenoids may play a protective role in depressive symptoms. First, depression may be linked to a lower expression of brain-derived neurotrophic factors. Interleukin-6 (IL-6) and tumor necrosis factor-α (TNF-α) levels were significantly increased in patients with depression. These inflammatory cytokines impair the expression of brain-derived neurotrophic factors, thus resulting in the occurrence of depression (33). Beta-carotene has been shown to reduce the mRNA levels of IL-6 and TNF-α (34). Zeaxanthin can also reduce the expression of IL-6, interleukin-1β (IL-1β), and TNF-α in the hippocampus (35). Second, the development of depression is related to oxidative and an imbalance between antioxidants and prooxidants (36). The brain is thought to be susceptible to oxidative stress because of its high oxygen consumption and high lipid levels (37). Forlenza and Miller (38) found increased serum levels of 8-hydroxy-2’-deoxyguanosine in clinical depression, which is a biological marker of DNA damage caused by oxidative stress, indicating that depression is accompanied by oxidative stress. Carotenoids are natural antioxidants that can effectively remove reactive oxygen species and other free radicals and protect organisms from oxidative damage (15). They are one of the most efficient physical quenchers of ^1^O_2_ because of their triplet energy levels lying close to that of ^1^O_2_. Carotenoids can scavenge free radicals by electron transfer between the free radical and carotenoid, forming a carotenoid radical cation or carotenoid radical anion. Free radicals can also be scavenged through hydrogen atom transfer, leading to a neutral carotenoid radical (39). The association between beta-carotene and depressive symptoms was U shaped. It is possible that when at high doses of beta-carotene, their ability to protect cells was lost, and the antioxidant capability would decrease or even become prooxidant activity (40). In addition, several nutrients and drugs could affect the bioavailability of carotenoids. For example, dietary fibers and minerals have been shown to impair the bioavailability of carotenoids. Other micronutrients may compete with carotenoids for absorption in the small bowel because they are usually consumed together. Fat absorption inhibitors, such as orlistat, could decrease the absorption of carotenoids (41). In summary, carotenoid can contribute to the prevention of depressive symptoms through anti-inflammatory and antioxidant capabilities.

Our study had several strengths. First, we explored the dose–response relationship between dietary carotenoid intakes and depressive symptoms. Second, our sample of subjects is large and representative based on NHANES. Third, we evaluated the associations between depressive symptoms with different kinds of dietary carotenoid, including alpha-carotene, beta-carotene, beta-cryptoxanthin, lycopene, lutein with zeaxanthin, and total carotenoid. Fourth, we adjusted for some potential confounding factors when exploring the associations and dose–response relationships between dietary carotenoid intakes and depressive symptoms.

However, our study has some limitations. First, causality cannot be determined because our study is a cross-sectional study. Second, although we have adjusted for some confounding factors, there may still be some unknown confounding factors left. Besides, PHQ-9 is a self-report scale instead of a diagnostic instrument for clinical depression, which can lead to misclassification. Fourth, the dietary data were obtained through two 24-h dietary recall interviews, which might have caused recall bias.

## Conclusion

In conclusion, our study suggests that alpha-carotene, beta-carotene, beta-cryptoxanthin, lycopene, lutein with zeaxanthin, and total carotenoid intakes were inversely associated with the risk of depressive symptoms among the U.S. adults. The dose–response relationship showed that these associations were non-linear. These findings need to be confirmed by prospective studies.

## Supplementary Material

Associations between dietary carotenoid intakes and the risk of depressive symptoms

Associations between dietary carotenoid intakes and the risk of depressive symptoms

## References

[1] World Health Organization Depression fact sheet. 2020 Available from: http://www.who.int/en/news-room/fact-sheets/detail/depression [cited 21 April 2019].

[2] World Health Organization Depression and other common mental disorders: global health estimates. Available from: http://apps.who.int/iris/bitstream/handle/10665/254610/WHO-MSD-MER-2017.2-eng.pdf?sequence=1&isAllowed=y [cited 21 April 2019].

[3] Mrazek DA, Hornberger JC, Altar CA, Degtiar I A review of the clinical, economic, and societal burden of treatment-resistant depression: 1996–2013. Psychiatr Serv 2014; 65(8): 977–87. doi: 10.1176/appi.ps.201300059 24789696

[4] Mathers CD, Loncar D Projections of global mortality and burden of disease from 2002 to 2030. PLoS Med 2006; 3(11): e442. doi: 10.1371/journal.pmed.0030442 17132052 PMC1664601

[5] Kramer IM, Simons CJ, Myin-Germeys I, Jacobs N, Derom C, Thiery E, et al. Evidence that genes for depression impact on the pathway from trauma to psychotic-like symptoms by occasioning emotional dysregulation. Psychol Med 2012; 42(2): 283–94. doi: 10.1017/S0033291711001474 21835094

[6] Nordquist N, Oreland L Serotonin, genetic variability, behaviour, and psychiatric disorders – a review. Ups J Med Sci 2010; 115(1): 2–10. doi: 10.3109/03009730903573246 20187845 PMC2853349

[7] Kaplow JB, Saunders J, Angold A, Costello EJ Psychiatric symptoms in bereaved versus nonbereaved youth and young adults: a longitudinal epidemiological study. J Am Acad Child Adolesc Psychiatry 2010; 49(11): 1145–54. doi: 10.1016/j.jaac.2010.08.004 20970702 PMC2965565

[8] Brydon L, Harrison NA, Walker C, Steptoe A, Critchley HD Peripheral inflammation is associated with altered substantia nigra activity and psychomotor slowing in humans. Biol Psychiatry 2008; 63(11): 1022–9. doi: 10.1016/j.biopsych.2007.12.007 18242584 PMC2885493

[9] Li F, Liu X, Zhang D Fish consumption and risk of depression: a meta-analysis. J Epidemiol Community Health 2016; 70(3): 299–304. doi: 10.1136/jech-2015-206278 26359502

[10] Wang L, Shen X, Wu Y, Zhang D Coffee and caffeine consumption and depression: a meta-analysis of observational studies. Aust N Z J Psychiatry 2016; 50(3): 228–42. doi:10.1177/0004867415603131 26339067

[11] Liu X, Yan Y, Li F, Zhang D Fruit and vegetable consumption and the risk of depression: a meta-analysis. Nutrition 2016; 32(3): 296–302. doi: 10.1016/j.nut.2015.09.009 26691768

[12] Xu H, Li S, Song X, Li Z, Zhang D Exploration of the association between dietary fiber intake and depressive symptoms in adults. Nutrition 2018; 54: 48–53. doi:10.1016/j.nut.2018.03.009 29747090

[13] Sun C, Wang R, Li Z, Zhang D Dietary magnesium intake and risk of depression. J Affect Disord 2019; 246: 627–32. doi: 10.1016/j.jad.2018.12.114 30611059

[14] Li Z, Wang W, Xin X, Song X, Zhang D Association of total zinc, iron, copper and selenium intakes with depression in the US adults. J Affect Disord 2018; 228: 68–74. doi: 10.1016/j.jad.2017.12.004 29232566

[15] Fiedor J, Burda K Potential role of carotenoids as antioxidants in human health and disease. Nutrients 2014; 6(2): 466–88. doi: 10.3390/nu6020466 24473231 PMC3942711

[16] Rubin LP, Ross AC, Stephensen CB, Bohn T, Tanumihardjo SA Metabolic effects of inflammation on vitamin A and carotenoids in humans and animal models. Adv Nutr 2017; 8(2): 197–212. doi: 10.3945/an.116.014167 28298266 PMC5347109

[17] Leoncini E, Edefonti V, Hashibe M, Parpinel M, Cadoni G, Ferraroni M, et al. Carotenoid intake and head and neck cancer: a pooled analysis in the International head and neck cancer epidemiology consortium. Eur J Epidemiol 2016; 31(4): 369–83. doi: 10.1007/s10654-015-0036-3 25930054 PMC5552068

[18] Bhupathiraju SN, Wedick NM, Pan A, Manson JE, Rexrode KM, Willett WC, et al. Quantity and variety in fruit and vegetable intake and risk of coronary heart disease. Am J Clin Nutr 2013; 98(6): 1514–23. doi: 10.3945/ajcn.113.066381 24088718 PMC3831537

[19] Bernstein PS, Li B, Vachali PP, Gorusupudi A, Shyam R, Henriksen BS, et al. Lutein, zeaxanthin, and meso-zeaxanthIn: the basic and clinical science underlying carotenoid-based nutritional interventions against ocular disease. Prog Retin Eye Res 2016; 50: 34–66. doi: 10.1016/j.preteyeres.2015.10.003 26541886 PMC4698241

[20] Li Z, Chen J, Zhang D Association between dietary carotenoid intakes and hypertension in adults: National Health and Nutrition Examination Survey 2007–2014. J Hypertens 2019; 37(12): 2371–79. doi: 10.1097/HJH.0000000000002200 31356404

[21] Coyne T, Ibiebele TI, Baade PD, Dobson A, McClintock C, Dunn S, et al. Diabetes mellitus and serum carotenoids: findings of a population-based study in Queensland, Australia. Am J Clin Nutr 2005; 82(3): 685–93. doi: 10.1093/ajcn.82.3.685 16155284

[22] Li D, Li Y Associations of alpha-carotenoid and beta-carotenoid with depressive symptoms in late midlife women. J Affect Disord 2019; 256: 424–30. doi: 10.1016/j.jad.2019.06.003 31229931

[23] Niu K, Guo H, Kakizaki M, Cui Y, Ohmori-Matsuda K, Guan L, et al. A tomato-rich diet is related to depressive symptoms among an elderly population aged 70 years and over: a population-based, cross-sectional analysis. J Affect Disord 2013; 144(1–2): 165–70. doi: 10.1016/j.jad.2012.04.040 22840609

[24] Park JY, You JS, Chang KJ Dietary taurine intake, nutrients intake, dietary habits and life stress by depression in Korean female college students: a case-control study. J Biomed Sci 2010; 17(Suppl 1): S40. doi: 10.1186/1423-0127-17-S1-S40 20804617 PMC2994399

[25] Payne ME, Steck SE, George RR, Steffens DC Fruit, vegetable, and antioxidant intakes are lower in older adults with depression. J Acad Nutr Diet 2012; 112(12): 2022–7. doi:10.1016/j.jand.2012.08.026 23174689 PMC3520090

[26] Prohan M, Amani R, Nematpour S, Jomehzadeh N, Haghighizadeh MH Total antioxidant capacity of diet and serum, dietary antioxidant vitamins intake, and serum hs-CRP levels in relation to depression scales in university male students. Redox Rep 2014; 19(3): 133–9. doi: 10.1179/1351000214Y.0000000085 24524538 PMC6837702

[27] Centers for Disease Control and Prevention National health and nutrition examination survey. Available from: http://www.cdc.gov/nchs/nhanes/index.htm [cited 20 April 2019].

[28] National Health and Nutrition Examination Survey Mental health – depression screener data documentation, codebook and frequencies. Available from: http://wwwn.cdc.gov/Nchs/Nhanes/2015-2016/DPQ_I.htm [cited 25 April 2019].

[29] Blanton CA, Moshfegh AJ, Baer DJ, Kretsch MJ The USDA automated multiple-pass method accurately estimates group total energy and nutrient intake. J Nutr 2006; 136(10): 2594–9. doi: 10.1093/jn/136.10.2594 16988132

[30] Moshfegh AJ, Rhodes DG, Baer DJ, Murayi T, Clemens JC, Rumpler WV, et al. The US Department of agriculture automated multiple-pass method reduces bias in the collection of energy intakes. Am J Clin Nutr 2008; 88(2): 324–32. doi: 10.1093/ajcn/88.2.324 18689367

[31] The United States Department of Agriculture (USDA) Dietary sources of nutrients. Available from: http://www.ars.usda.gov/ba/bhnrc/fsrg [cited 21 August 2020].

[32] Center for Disease Control and Prevention National health and nutrition examination survey. Modul 3: weighting. Available from: http://wwwn.cdc.gov/nchs/nhanes/tutorials/module3.aspx [cited 21 April 2019].

[33] Numakawa T, Richards M, Nakajima S, Adachi N, Furuta M, Odaka H, et al. The role of brain-derived neurotrophic factor in comorbid depression: possible linkage with steroid hormones, cytokines, and nutrition. Front Psychiatry 2014; 5: 136. doi: 10.3389/fpsyt.2014.00136 25309465 PMC4175905

[34] Kim NR, Kim HY, Kim MH, Kim HM, Jeong HJ Improvement of depressive behavior by Sweetme Sweet Pumpkin and its active compound, beta-carotene. Life Sci 2016; 147: 39–45. doi: 10.1016/j.lfs.2016.01.036 26820672

[35] Zhou X, Gan T, Fang G, Wang S, Mao Y, Ying C Zeaxanthin improved diabetes-induced anxiety and depression through inhibiting inflammation in hippocampus. Metab Brain Dis 2018; 33(3): 705–11. doi: 10.1007/s11011-017-0179-x 29290042

[36] Sarandol A, Sarandol E, Eker SS, Erdinc S, Vatansever E, Kirli S Major depressive disorder is accompanied with oxidative stress: short-term antidepressant treatment does not alter oxidative-antioxidative systems. Hum Psychopharmacol 2007; 22(2): 67–73. doi: 10.1002/hup.829 17299810

[37] Menard C, Hodes GE, Russo SJ Pathogenesis of depression: insights from human and rodent studies. Neuroscience 2016; 321: 138–62. doi: 10.1016/j.neuroscience.2015.05.053 26037806 PMC4664582

[38] Forlenza MJ, Miller GE Increased serum levels of 8-hydroxy-2’-deoxyguanosine in clinical depression. Psychosom Med 2006; 68(1): 1–7. doi: 10.1097/01.psy.0000195780.37277.2a 16449405

[39] Krinsky NI Antioxidant functions of carotenoids. Free Radic Biol Med 1989; 7(6): 617–35. doi: 10.1016/0891-5849(89)90143-3 2695406

[40] Eghbaliferiz S, Iranshahi M Prooxidant activity of polyphenols, flavonoids, anthocyanins and carotenoids: updated review of mechanisms and catalyzing metals. Phytother Res 2016; 30(9): 1379–91. doi: 10.1002/ptr.5643 27241122

[41] Desmarchelier C, Borel P Overview of carotenoid bioavailability determinants: from dietary factors to host genetic variations. Trends Food Sci Technol 2017; 69: 270–80. doi: 10.1016/j.tifs.2017.03.002

