# Supplementary material for: Associations between dietary carotenoid intakes and the risk of depressive symptoms
Source: Food Nutr Res. 2020 Dec 28;64:10.29219/fnr.v64.3920. doi: 10.29219/fnr.v64.3920 (PMC7778430; doi:10.29219/fnr.v64.3920)
Supplement: Associations between dietary carotenoid intakes and the risk of depressive symptoms [file FNR-64-3920-s001.docx]

| **Supplementary Table S1.** Weighted odds ratios (95% confidence intervals) of depressive symptoms across quartiles of adjusted dietary carotenoid intakes, NHANES 2009-2016 (N =17401) | | | | |
| --- | --- | --- | --- | --- |
|  | Case/Participants | Crude1^a^ | Model 1^a^ | Model 2^a^ |
| Alpha-carotene (mcg/Kg/day) |  |  |  |  |
| <0.29 | 536/4348 | 1.00 (Ref.) | 1.00 (Ref.) | 1.00 (Ref.) |
| 0.29 to <1.07 | 376/4335 | 0.63(0.50-0.70)** | 0.63(0.50-0.79)** | 0.78(0.61-1.00) |
| 1.07 to <5.87 | 337/4297 | 0.61(0.49-0.75)** | 0.58(0.48-0.72)** | 0.73(0.57-0.93)* |
| ≥5.87 | 281/4288 | 0.48(0.38-0.60)** | 0.46(0.37-0.58)** | 0.68(0.52-0.88)** |
| Beta-carotene (mcg/Kg/day) |  |  |  |  |
| <5.24 | 573/4322 | 1.00 (Ref.) | 1.00 (Ref.) | 1.00 (Ref.) |
| 5.24 to <13.30 | 401/4316 | 0.62(0.50-0.76)** | 0.61(0.49-0.76)** | 0.69(0.54-0.87)** |
| 13.30 to <34.92 | 298/4313 | 0.47(0.37-0.59)** | 0.46(0.37-0.58)** | 0.59(0.45-0.77)** |
| ≥34.92 | 256/4313 | 0.39(0.32-0.48)** | 0.37(0.30-0.46)** | 0.57(0.45-0.73)** |
| Beta-cryptoxanthin (mcg/Kg/day) |  |  |  |  |
| <0.17 | 470/4348 | 1.00 (Ref.) | 1.00 (Ref.) | 1.00 (Ref.) |
| 0.17 to <0.53 | 437/4344 | 0.93(0.73-1.19) | 0.92(0.72-1.18) | 1.04(0.80-1.35) |
| 0.53 to <1.29 | 331/4299 | 0.69(0.55-0.87)** | 0.69(0.55-0.87)** | 0.78(0.60-1.02) |
| ≥1.29 | 291/4277 | 0.56(0.43-0.72)** | 0.55(0.43-0.71) * | 0.75(0.56-0.99)* |
| Lycopene (mcg/Kg/day) |  |  |  |  |
| <9.05 | 460/4322 | 1.00 (Ref.) | 1.00 (Ref.) | 1.00 (Ref.) |
| 9.05 to <31.00 | 386/4317 | 0.82(0.66-1.02) | 0.82(0.65-1.03) | 0.90(0.71-1.14) |
| 31.00 to <80.04 | 382/4317 | 0.86(0.66-1.12) | 0.86(0.66-1.13) | 0.97(0.72-1.30) |
| ≥80.04 | 300/4317 | 0.55(0.42-0.72)** | 0.56(0.42-0.74)** | 0.68(0.50-0.93)* |
| Lutein with zeaxanthin (mcg/Kg/day) |  |  |  |  |
| <5.51 | 566/4317 | 1.00 (Ref.) | 1.00 (Ref.) | 1.00 (Ref.) |
| 5.51 to <10.28 | 410/4321 | 0.77(0.65-0.91)** | 0.78(0.66-0.92)** | 0.88(0.71-1.09) |
| 10.28 to <20.28. | 317/4313 | 0.53(0.42-0.67)** | 0.54(0.43-0.68)** | 0.68(0.54-0.87)** |
| ≥20.28 | 235/4313 | 0.34(0.27-0.43)** | 0.32(0.25-0.41)** | 0.49(0.37-0.64)** |
| Total carotenoid (mcg/Kg/day) |  |  |  |  |
| <37.34 | 544/4316 | 1.00 (Ref.) | 1.00 (Ref.) | 1.00 (Ref.) |
| 37.34 to <80.38 | 394/4316 | 0.77(0.63-0.95)* | 0.80(0.65-0.93)* | 0.91(0.74-1.13) |
| 80.38 to <157.18 | 322/4316 | 0.56(0.46-0.68)** | 0.55(0.45-0.67)** | 0.70(0.55-0.88)** |
| ≥157.18 | 268/4316 | 0.42(0.33-0.54)** | 0.43(0.34-0.54)** | 0.64(0.48-0.86)** |
| ^a^Calculated using binary logistic regression. Model 1 adjusted for age and gender. Model 2 adjusted for age and gender, ethnicity, educational level, BMI, annual family income, work activity, recreational activity, hypertension, diabetes, smoking status, drinking status and total energy intake. *p < 0.05; **p < 0.01. | | | | |
