# Supplementary material for: Associations between dietary carotenoid intakes and the risk of depressive symptoms
Source: Food Nutr Res. 2020 Dec 28;64:10.29219/fnr.v64.3920. doi: 10.29219/fnr.v64.3920 (PMC7778430; doi:10.29219/fnr.v64.3920)
Supplement: Associations between dietary carotenoid intakes and the risk of depressive symptoms [file FNR-64-3920-s002.docx]

| **Supplementary Table S2.** Weighted odds ratios (95% confidence intervals) of depressive symptoms across quartiles of dietary total carotenoid intake, stratified by ethnicity, NHANES 2009-2016 (N =17401) | | | |
| --- | --- | --- | --- |
| Total carotenoid | Crude^a^ | Model 1^a^ | Model 2^a^ |
| Mexican American |  |  |  |
| <3088 | 1.00 (Ref.) | 1.00 (Ref.) | 1.00 (Ref.) |
| 3088 to <6380 | 0.91(0.58-1.43) | 0.94(0.59-1.50) | 0.81(0.46-1.42) |
| 6380 to <12179 | 0.71(0.44-1.15) | 0.73(0.45-1.18) | 0.79(0.42-1.48) |
| ≥12179 | 0.73(0.38-1.37) | 0.78(0.42-1.45) | 0.94(0.47-1.89) |
| Other Hispanic |  |  |  |
| <3088 | 1.00 (Ref.) | 1.00 (Ref.) | 1.00 (Ref.) |
| 3088 to <6380 | 0.99(0.63-1.54) | 1.02(0.65-1.60) | 1.21(0.70-2.08) |
| 6380 to <12179 | 0.56(0.33-0.95)* | 0.60(0.34-1.05) | 0.67(0.36-1.23) |
| ≥12179 | 0.50(0.30-0.83)** | 0.55(0.32-0.94)* | 0.55(0.28-1.09) |
| Non-Hispanic white |  |  |  |
| <3088 | 1.00 (Ref.) | 1.00 (Ref.) | 1.00 (Ref.) |
| 3088 to <6380 | 0.59(0.43-0.82)** | 0.62(0.44-0.87)** | 0.72(0.51-1.01) |
| 6380 to <12179 | 0.53(0.40-0.71)** | 0.54(0.41-0.72)** | 0.71(0.52-0.97)* |
| ≥12179 | 0.35(0.25-0.50)** | 0.37(0.26-0.53)** | 0.57(0.39-0.85)** |
| Non-Hispanic black |  |  |  |
| <3088 | 1.00 (Ref.) | 1.00 (Ref.) | 1.00 (Ref.) |
| 3088 to <6380 | 0.83(0.57-1.20) | 0.83(0.58-1.20) | 0.82(0.56-1.20) |
| 6380 to <12179 | 0.85(0.51-1.43) | 0.85(0.51-1.42) | 0.77(0.44-1.34) |
| ≥12179 | 0.59(0.40-0.88)** | 0.62(0.42-0.91)* | 0.68(0.42-1.10) |
| Other ethnicity |  |  |  |
| <3088 | 1.00 (Ref.) | 1.00 (Ref.) | 1.00 (Ref.) |
| 3088 to <6380 | 0.95(0.32-2.80) | 0.95(0.33-2.66) | 0.78(0.30-2.01) |
| 6380 to <12179 | 0.65(0.29-1.42) | 0.65(0.30-1.43)** | 0.64(0.25-1.68) |
| ≥12179 | 0.43(0.24-0.77)** | 0.42(0.23-0.77) | 0.42(0.20-0.89)* |
| ^a^Calculated using binary logistic regression. Model 1 adjusted for age and gender. Model 2 adjusted for age and gender, educational level, BMI, annual family income, work activity, recreational activity, hypertension, diabetes, smoking status, drinking status and total energy intake. *p < 0.05; **p < 0.01. | | | |
